# Retraction Note: Robust method for TALEN-edited correction of pF508del in patient-specific induced pluripotent stem cells

**DOI:** 10.1186/s13287-017-0591-5

**Published:** 2017-06-02

**Authors:** María Vicenta Camarasa, Víctor Miguel Gálvez

**Affiliations:** Caubet-Cimera Foundation, Hospital Joan March, Ctra Soller Km 12, 07110 Bunyola, Mallorca Spain

## Retraction Note

This article [1] has been retracted by the Editor because the authors do not have ownership of the data they report. A formal investigation conducted by the Consejo Superior de Investigaciones Científicas (CSIC) and Fundación de Investigación Sanitaria de las Islas Baleares Ramon Llull (FISIB) has concluded that the data reported in this article are the sole property of the Consejo Superior de Investigaciones Científicas (CSIC) and the Fundación de Investigación Sanitaria de las Islas Baleares Ramon Llull (FISIB). The authors do not agree with this retraction.

## References

[1] Camarasa MV, Gálvez VM (2016). Robust method for TALEN-edited correction of pF508del in patient-specific induced pluripotent stem cells. Stem Cell Res Ther.

